# Versatile bipolar temperature controller for custom *in vitro* applications

**DOI:** 10.1016/j.ohx.2020.e00155

**Published:** 2020-11-03

**Authors:** Sabrina Asteriti, Lorenzo Cangiano

**Affiliations:** a Dept. of Translational Research, University of Pisa, Pisa, Italy; b Dept. of Neurosciences, Biomedicine and Movement Sciences, University of Verona, Verona, Italy

**Keywords:** Heating, Cooling, *In vitro*, Control system, Temperature, Electrophysiology

## Abstract

Effective temperature control is crucial in many studies of isolated biological tissues, with preparations often requiring specialized holding chambers. In these situations, the design flexibility and optimizations offered by a custom made temperature controller may be preferable over a commercial model. We present a versatile controller for heating and cooling applications, providing simple step-by-step instructions to mathematically model your specific system and optimize controller parameters. The apparatus uses analog components and linear stages to simplify circuit comprehension and customization, achieving fast transitions with small static errors and overshoots over a wide range of temperatures without readjustment. A fully featured rackable enclosure is complemented by two temperature probes based on the LMT70A linear microchip sensor (for the control loop and for bath monitoring). BNC outputs provide scaled probe signals for continuous temperature data acquisition. The maximum achievable power output of the controller is −23.5 W/+22.0 W (−4.7 V/+4.4 V, ±5.0 A), sufficient to bring a well designed holder for standard 35 mm chambers from 23 °C up to 37 °C in ~1 min and down to 3 °C in ~4 min. Any biologist with some technical prowess should be able to follow our instructions from modeling to assembly and calibration.

## Highlights

• Effective temperature control is crucial in many *in vitro* tissue studies.
• Electrophysiology or imaging often require specialized holding chambers.
• Our custom temperature controller offers versatility and highly optimized operation.
• Cooling and heating provide fast temperature changes over a wide physiological range.
• Step-by-step simple but theoretically rigorous instructions enable user optimization.

## Graphical abstract

**Figure:**
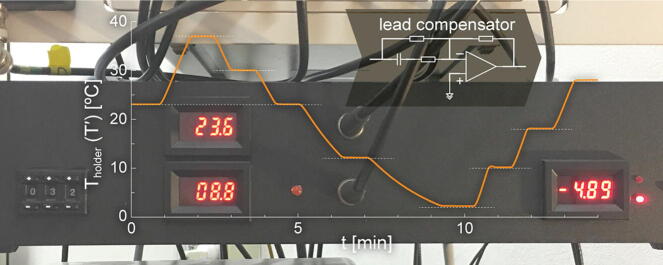

Specifications table

**Table:** 

Hardware name	VersatileBTC 1
Subject areas	· Medical · Neuroscience · Biological Sciences
Hardware type	· Biological sample handling and preparation
Open Source License	Attribution-ShareAlike 4.0 International (CC BY-SA 4.0)
Cost of Hardware	*1029€*
Source File Repository	*Open Science Framework (OSF)*

## 1 Hardware in context

Effective temperature control of biological tissues is a crucial aspect in *in vitro* studies [1]. Physiological temperatures vary from a few degrees above 0 °C in many aquatic animals to around 37 °C in mammals and span wide ranges in invertebrates. Temperature often has a profound impact on cellular and network functional properties, as exemplified by the visual system [2], [3], [4]. The restricted space available around tissue chambers poses a challenge for effective thermal control [5]. For example, mammalian slices require vigorous superfusion with oxygenated solution to maintain acceptable O_2_ partial pressure levels [6]. Thus, inflow/outflow rate must be kept high relative to bath volume. When using upright microscopes water immersion objectives represent another important source of thermal perturbation. Furthermore, in electrophysiological experiments the chamber must be open to ambient air to allow electrode access. The main strategy to tackle these problems is to clamp the chamber holder at a temperature adequate to maintain the desired temperature in the chamber, with both components designed so as to minimize mismatches. The inflowing solution, if present, should also be conditioned. *Ad hoc* tissue holding chambers may be necessary for some preparations. Another approach is to attempt to directly clamp the temperature of the bath: this, unfortunately, is very problematic for reasons that we discuss in Section 2.7. In summary, the canonical controller should be able to maintain a tight control on holder temperature, while providing a reading of the bath temperature. In electrophysiology an additional requirement is that of avoiding introducing any noise in the recordings, for instance due to switching components in the controller circuitry. Commercial models (see table for some examples) generally provide very little information on their control properties or internal components. Therefore, in many situations the design flexibility and optimizations offered by a custom made temperature controller may be preferable over a general purpose commercial model. Our main goals for a custom controller for electrophysiology were: (*i*) versatility for both heating and cooling applications; (*ii*) rapid responses, minimal overshoot and small static error; (*iii*) simple to use (i.e. able to cover a wide temperature range without readjustments); (*iv*) minimization of potential noise contributions during recordings; (*v*) ease of additional customization. We based our design on the LMT70A, an active microchip temperature sensor with a steep and essentially-linear response in the range of interest [7] (see Section 2.6). A non-switched actuator circuit [8] is used to drive two thermoelectric modules (TEMs), electrically in series but thermally in parallel. Power is provided by a remotely located linear supply. The maximum achievable output of the controller is −23.5 W/+22.0 W with 50 Hz mains (−4.7 V/+4.4 V and ± 5.0 A) (12% more current and power are available with 60 Hz mains), sufficient to bring a well designed tissue chamber holder for standard 35 mm dishes from 23 °C up to 37 °C in about 1 min and down to 3 °C in 4 min. Low resistance TEMs should be used given this output envelope, something to keep in mind when matching this controller with a custom or commercial holder.

Commented selection of commercial temperature controllers

**Table:** 

Brand and model	Cost (approx., excl. VAT)	Comments
***VersatileBTC 1 (this controller)***	***€1029***	***(i) Lead compensation control (PD-type) with step-by-step modeling-based instructions on how to maximize response speed and minimize static error. (ii) Sensors and temperature selector can be calibrated. (iii) Guaranteed lowest noise linear power supply and actuator stage. (iv) Output power lower than commercial models but more than sufficient to enact rapidly changes in temperature of a patch-clamp type holder. (v) Additional features easy to add.***
*npi electronic PTC-10*	*€2410*	*(i) Proportional-integral PI control with no derivative/lead compensation ⇒ much slower response and potential stability issues; the zero static error offered by PI control is unlikely to be beneficial in experiments, as the bath temperature will unavoidably suffer significant environmental perturbations anyhow; user likely required to tweak gain, integration time constant and current limits with every change in desired temperature. (ii) Sensor calibration not accessible. (iii) Higher output power (52 W continuous) likely obtained with a switching supply (not specified). (iv) Type of actuator circuit not specified.*
*ALA Scientific HCT-10*	*€2205*	*(i) Uses PID control but the user is only allowed to select slow/medium/fast response ⇒ optimal control with fast responses and minimal overshoots unlikely to be attainable. (ii) Sensor calibration not accessible. (iii) Type of power supply and actuator circuit not specified.*
*Dagan Corporation HCC-100A*	*$4550*	*(i) Proportional-integral PI control with no derivative/lead compensation ⇒ much slower response and potential stability issues; user cannot tweak feedback loop parameters. (ii) Sensor calibration not accessible. (iii) High output power (80 W) likely obtained with a switching supply (not specified); V and I limits not stated. (iv) Type of actuator circuit not specified.*
*Luigs & Neumann TC07*	*€2001*	*(i) PID controller with parameters pre-optimized for the holders from the same company; complex trial-and-error parameter customization by the user possible via software. (ii) Sensor calibration not accessible. (iii) High output power (90 W) likely obtained with a switching supply (not specified). (iv) Type of actuator circuit not specified.*
*Warner Instruments CL-100*	*€2895*	*(i) Controller type not specified; the user is only allowed to select slow/medium/fast response ⇒ optimal control with fast responses and minimal overshoots unlikely to be attainable. (ii) Sensor calibration not accessible. (iii) Type of power supply and actuator circuit not specified.*

## 2 Hardware description

**Fig. 1:**
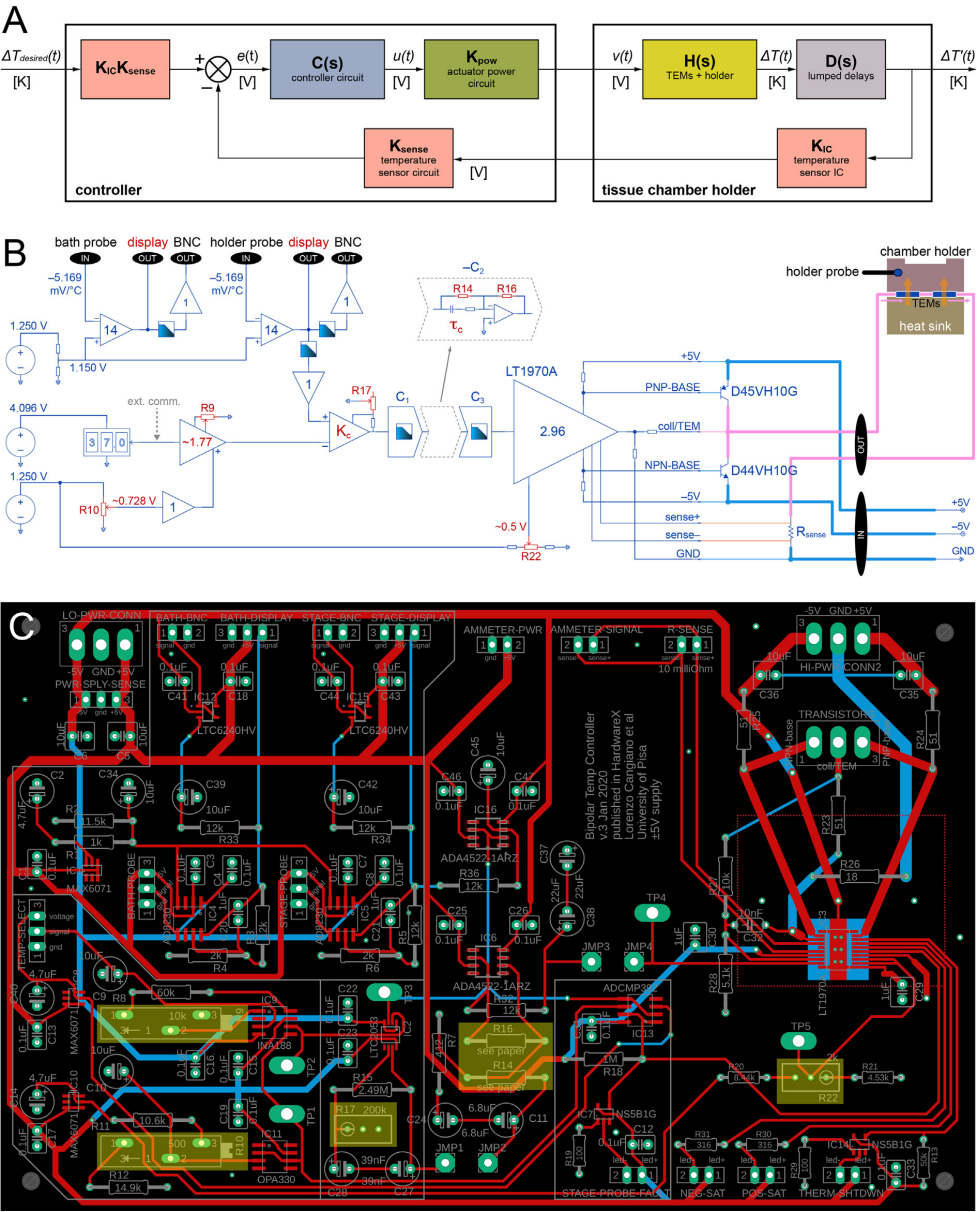
Simplified system diagram and circuit schematic. (A) Block diagram of the feedback loop consisting of the controller chassis, tissue chamber holder and temperature probe. Block behavior is described by transfer functions defined in the complex-domain and derived in a small signals approximation. In the frequency range relevant to temperature control (up to a few Hz) the blocks labelled K_…_ behave as real constants. Connecting arrows are labelled with the name of the time-domain variable used in the analysis and its units. (B) Simplified schematic of the backstage circuitry, power transistors and chamber holder. Here two TEMs are connected electrically in series but thermally in parallel, such that they pump heat in the same direction (only the example of the holder being heated is shown). Components in red: calibration required during assembly; thick lines: wires that must be able to handle 5–6 A with minimal voltage drops; circles: precision voltage references; triangles: amplifiers (labelled with their gains); shaded boxes: low pass filters; black ovals: inputs/outputs; small rectangles: fixed resistors; small rectangles with arrows: variable resistors; dashed polygon: the key lead compensator stage; ext. comm.: see Section 8.2 (C) Actual backstage printed circuit board. Calibration points are highlighted in yellow. Red traces: top layer; blue traces: bottom layer. Copper fills for the ground plane are not shown. (For interpretation of the references to colour in this figure legend, the reader is referred to the web version of this article.)

Conceptually, the control loop is as follows (Fig. 1): the user selects the desired temperature on the controller chassis; an error signal is generated by comparing the desired and actual temperatures; this signal is amplified and modulated in time to achieve optimal control; a push-pull power actuator stage drives the TEMs; these pump heat to/from the chamber holder; heat conduction between the TEMs and the temperature sensor introduces time and phase delays; a signal is sent back to the chassis where it is scaled and fed to the comparator. Here we provide a brief summary of each block and present practical feedback loop optimization instructions for user customization. For small changes of the desired temperature each block can be linearized and described by a transfer function in the s-domain. Detailed derivations (provided only for completeness) and functions/plots in CDF public format (executable in Mathematica® or the free Wolfram Player) can be found in the design files. Even the reader and prospective builder without any knowledge of complex-domain mathematics or control theory should be able to follow all the steps, including simulating her/his system to find optimal controller parameters.

### 2.1 H(s)– TEMs and tissue chamber holder assembly

Since TEMs are more efficient in heating mode, their behavior is best examined in this condition [9] (in cooling mode the open loop gain will be lower and thus the system guaranteed to be stable, albeit with a larger static error). The cold side of the TEMs is clamped at ambient temperature by a heat sink (our system uses water cooling). The hot side of the TEMs and the chamber holder are assumed to be isothermal. The TEMs must be in series electrically but in parallel thermally. Linearization around the desired temperature (see *transfer function derivations.pdf*) leads to the transfer function:

(1) $$H\left(s\right)=\frac{\Delta T(s)}{\Delta V(s)}=\frac{K_{s}}{1+\tau_{s}s}$$

Thus, the time-domain response of this block to a unitary step in drive voltage is simply:

$$\Delta T\left(t\right)=K_{s}\left(1-e^{-t/\tau_{s}}\right)$$

where $\tau_{s}$ is the single thermal time constant of the assembly and $K_{s}$ its static gain. Both parameters could be estimated from the TEMs’ datasheet and the chamber holder properties (*transfer function derivations.pdf* and Eqs. (23)–(25) in [10]). However, it is simpler to ignore $K_{s}$ (it is absorbed in the overall open loop gain of the system) and to measure $\tau_{s}$: (*i*) heat the chamber holder to, say, 37 °C; (*ii*) power off the TEMs; (*iii*) record the decay in temperature and determine its time constant. In our custom holder $\tau_{s}=198\text{s}$.

### 2.2 D(s)– Lumped thermal delays

Thermal conduction between the TEMs and the temperature sensor introduces small time and phase delays. Examining the temperature response of our system to a step change in TEM drive (open loop configuration) we found that these delays are well described by the transfer function:

(2) $$D\left(s\right)=\frac{1-\frac{t_{0}}{2}s}{1+\frac{t_{0}}{2}s}\cdot \frac{1}{1+\tau_{d}s}$$

The first term is a rational polynomial approximation of a fixed time delay $t_{0}$, while the second is a first order low pass filter with time constant $\tau_{d}$. In our custom holder $t_{0}=0.8\text{s}$, $\tau_{d}=5.5\text{s}$.

### 2.3 K_IC_, K_sense_, K_pow_ – Temperature sensor circuitry and power actuator

In the frequency range relevant to temperature control (up to a few Hz) these blocks behave simply as real multiplicative terms (at higher frequencies signals are suppressed by low pass filters present in the controller). They can thus be ignored since they are absorbed in the overall open loop gain of the system. In any case their values are *K_IC_* = −5.169 mV/ °C (with two point calibration, 5.4, 5.5) [7], *K_sense_* = −14 and *K_pow_* = 2.96 (Fig. 1B).

### 2.4 Overall system transfer function

From Fig. 1 the open loop transfer function of the system is:

(3) $$G\left(s\right)=K_{\mathrm{IC}}K_{\mathrm{sense}}C\left(s\right)K_{\mathrm{pow}}H\left(s\right)D\left(s\right)=KC\left(s\right)\frac{\left(1-\frac{t_{0}}{2}s\right)}{\left(1+\tau_{s}s\right)\left(1+\frac{t_{0}}{2}s\right)\left(1+\tau_{d}s\right)}$$

where $K:=K_{\mathrm{IC}}K_{\mathrm{sense}}K_{\mathrm{pow}}K_{s}$ is a dimensionless real constant. A simplification can be made where the two sensor-related blocks are moved to the forward path, resulting in a unity feedback loop. The closed loop transfer function is thus:

(4) $$G_{0}\left(s\right)=\frac{G(s)}{1+G(s)}$$

#### 2.4.1 C(s) – Performance with a proportional controller

**Fig. 2:**
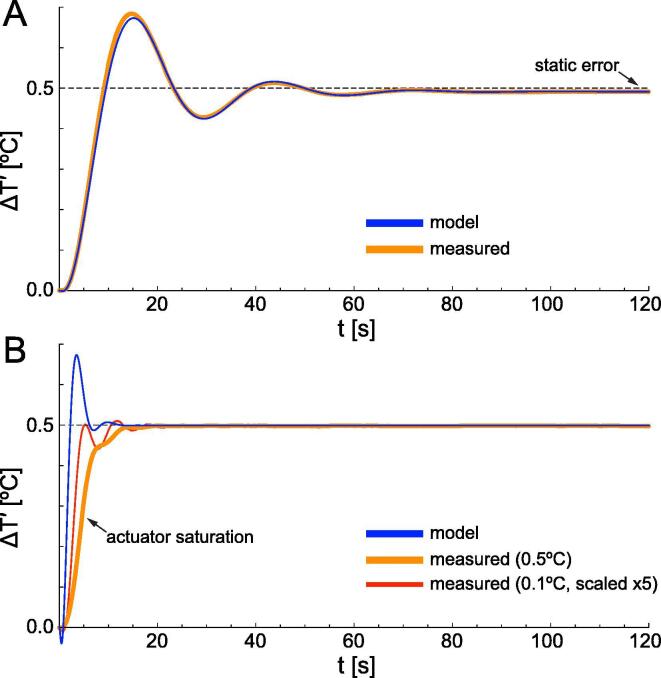
Response of our system to changes in desired temperature. (A) Response to a 0.5 °C step increase, starting from 37 °C, with a proportional controller. Measured (orange trace) or predicted by Eqs. (1)–(4) with parameters $K_{c}K=58;\tau_{s}=198\text{s};t_{0}=0.8\text{s};\tau_{d}=5.5\text{s}$ (*r1[s_]* in *transfer function derivations.pdf*) (blue trace). (B) Response to the same step when using lead compensation. Measured (orange trace) or predicted with parameters $K_{c}K=250;\tau_{s}=198\text{s};t_{0}=0.8\text{s};\tau_{d}=5.5\text{s};\tau_{c}=3.17\text{s}$ (*r2[s_]*) (blue trace). Note that the higher gain $K_{c}K$ now used briefly saturates the actuator circuit, leading to a slower rise and lack of ringing in the measured response. This is confirmed by examining the scaled response to a smaller 0.1 °C step (red trace): a shorter permanence in saturation better replicates theory. (For interpretation of the references to colour in this figure legend, the reader is referred to the web version of this article.)

The last step in system design is selecting an optimal control strategy and tweaking its parameters for the user’s conditions. After building the actual system (Section 5) we tested performance with a quasi-proportional controller $C\left(s\right)=K_{c}C_{1}\left(s\right)\approx K_{c}$ (proportional, except for some low pass filtering; see $C_{1}\left(s\right)$ in Eq. (5)). We increased $K_{c}$ until some ringing began to appear in the temperature response of the system (Fig. 2A, orange trace) and measured a static error of 1.5%. Since the latter is given by $100\%/(1+K_{c}K)$, we estimated the overall open loop gain $K_{c}K$ of our system at ~ 65. From Eqs. (1) to (4) we computed the response to a small change in desired temperature (*r1[s_]* in *modeling.cdf*) and found an excellent match to experimental behavior when using $K_{c}K=58$ (Fig. 2A, blue trace). Therefore, Eqs. (1)–(4) adequately describe this system and can be used to model an optimized controller.

#### 2.4.2 C(s) – Performance with a lead compensator controller

**Fig. 3:**
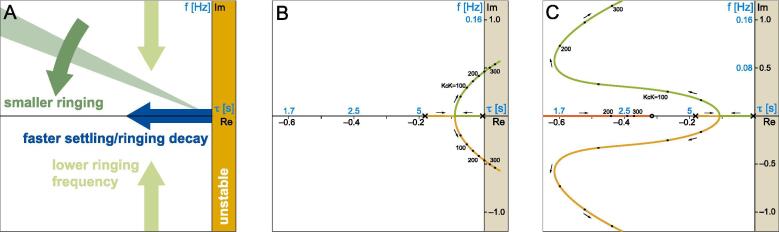
Inspection of the *root locus plot* (RLP) of the feedback loop is key to controller optimization. (A) Effect of root position in the complex plane on system response. Ringing frequency is shown on the y-axis (blue units), while settling/oscillation decay time constant is shown on the x-axis (blue units). (B) RLP for our system when using a quasi-proportional controller $C\left(s\right)=K_{c}C_{1}\left(s\right)$. Each of the two colored branches shows the trajectory of a root as the open loop gain $K_{c}K$ varies from zero (✕) to several hundreds. (C) RLP for the lead compensation controller $C\left(s\right)=K_{c}C_{1}\left(s\right)C_{2}\left(s\right)C_{3}\left(s\right)$ with $\tau_{c}=3.17\text{s}$. Three dominant roots are now present, which in a wide range of gains lie to the left of those in B, leading to an improved transient response. In both B and C more roots lie to the left of the visualized range but have no significant impact as they represent very fast settling components. (For interpretation of the references to colour in this figure legend, the reader is referred to the web version of this article.)

To increase settling speed and reduce static error (see Section 2.5) we added a ‘lead compensator’ network $C_{2}\left(s\right)$ [11], followed by a low pass filter $C_{3}\left(s\right)$:

(5) $$C\left(s\right)=K_{c}C_{1}\left(s\right)C_{2}\left(s\right)C_{3}\left(s\right)=K_{c}\cdot \frac{1+\frac{0.05}{K_{c}}s}{1+0.05s}\cdot \frac{1+\tau_{c}s}{1+\alpha \tau_{c}s}\cdot \frac{1}{1+0.13s}$$

where α<0.001. The optimal time constant $\tau_{c}$ of the lead compensator depends on the user’s TEMs and chamber holder. It can be found either empirically or using our provided CDF code to examine the *root locus plot* (RLP) of the system (i.e. the location in the complex plane of the roots of the characteristic equation 1+G(s)=0, as a function of open loop gain $K_{c}K$). One should aim to bring the roots as left and near the real axis as possible (Fig. 3A). The RLP for the quasi-proportional controller (Fig. 3B; *p1* in *modeling.cdf*) explains the slow settling time and ringing observed with even small gains (Fig. 2A). Above 250 the system would become unstable. On the other hand, with lead compensation and an optimal (for our specific system) $\tau_{c}=3.17\text{s}$, much higher gains can be used with faster settling and acceptable ringing (Fig. 3C; *p2* in *modeling.cdf*). The actual performance of the controller with a gain of 250 (Fig. 2B, orange trace) tracks theory (blue trace) but lacks the initial overshoot due to a transient saturation of the power actuator (see figure legend).

### 2.5 Choice of compensation strategy

A lead compensator is an approximate, but circuit-wise simpler (Fig. 1B, inset), implementation of an ideal proportional-derivative (PD) controller. This generates a drive signal that is the sum of two components: one proportional to the error $K_{p}e$ and the other to its time derivative $K_{d}\overset{˙}{e}$, where $K_{p},K_{d}>0$. In our present case the error is the difference between the desired and current temperature. Let’s assume that the current temperature is below the desired one and it is increasing, thus e>0 and $\overset{˙}{e}<0$. The P term will be positive and large, contributing to further heating of the chamber holder by the TEMs. However, since the error is decreasing the D term will be negative and partially counteracting P. This counteraction does not play an important role until the error approaches zero: when this occurs D dominates over P resulting in a negative drive to the TEMs, which switch from heating to cooling the holder in anticipation (i.e. in ‘lead’) of the impending temperature overshoot (itself due to the delays in the system). The process continues resulting in smaller and more rapidly decaying ringing compared to a simple P controller. The last key point is that, due to the increased stability offered by PD control, $K_{p}$ can be safely increased to reduce the static error of the system. We could have complicated things by adding an integral term (e.g. with a lead-lag compensator) to bring the static error to zero. Two considerations argue against such a choice: (*i*) feedback loop stability would be sensitive to parameter selection by the user, and (*ii*) clamping of the chamber holder temperature to much below ± 0.1 °C is unnecessary, as the bath temperature is unavoidably subject to much larger fluctuations.

### 2.6 Choice of electronic components

Three desirable characteristics of any apparatus used for electrophysiology are reliability, accuracy and low noise. We selected the highest quality components for the controller electronics, with cost savings taking a low priority. The signal chain was designed to minimize drift by using high-precision, low-noise, zero or low-drift voltage references, amplifiers and resistors. We avoided using switching components, opting instead for graded output including a linear power supply and a linear power actuator [8]. Here we traded power efficiency for quiet operation, the objective being to avoid any potential contamination of recorded biological signals. Furthermore, non-switched control of TEMs contributes to their reliability [12]. The linear power supply is housed in a separate enclosure due to its significant size and to provide good passive cooling (Fig. 5). The two temperature probes are based on the Texas Instruments LMT70A miniature sensor [7]. Its advantages compared to a classic thermistor are twofold: (*i*) an essentially linear output between 0 °C and 37 °C; with two-point calibration at these physiological extremes, the expected inaccuracy due to non-linearity at the midpoint temperature of 18.5 °C is −0.04 °C (see LUT on p.20 in [7]); (*ii*) a measurement variation of ± 0.05 °C (max ± 0.15 °C) between microchips of the same batch. These characteristics enable not only simple two-point calibration and linear processing of the sensor signal, but plug-and-play exchangeability of probes. Furthermore, the LMT70A datasheet shows no evidence of internal switched operation that may generate noise.

### 2.7 Optimal chamber holder design

The reader planning to develop a custom chamber holder for our controller should be aware of the impact of the holder-specific parameters $\tau_{s},K_{s}$ (Eq. (1)) and $\tau_{0},\tau_{d}$ (Eq. (2)) on system performance. First, maximizing the holder’s mass/thermal dispersion ratio increases $\tau_{s}$ (Eq. (12) in *transfer function derivations.pdf*). This is beneficial as it improves stability, enabling the use of a higher open loop gain for a smaller static error. Second, minimizing thermal dispersion increases the holder’s static gain $K_{s}$ (see Eq. (11) in *transfer function derivations.pdf*). This is also very helpful, as the contribution of the controller to the overall open loop gain can be reduced, thereby limiting actuator saturation with improvements in response speed. Third, the delays $\tau_{0},\tau_{d}$ must be kept as small as possible: they dramatically deteriorate system stability and response speed. Incidentally, this is the reason why inserting the bath probe in the control loop is generally a bad idea. In summary, aim for a compact holder, insulated as much as possible from the environment, and intimately couple the temperature probe to the holder body (Figs. 1B, 6E).

In selecting compatible TEMs, which should be connected electrically in series but thermally in parallel, bear in mind that the power actuator stage max achievable output voltage (at coll/TEM in Fig. 1B) is −4.7 V/+4.4 V and the maximum deliverable output current is ± 5.0 A (50 Hz) or ± 5.6 A (60 Hz). These limits are dictated by the linear power supply and can be approached safely (official specifications are 7% higher, but power use by the backstage PCB must be also considered). TEMs operate most efficiently when strongly derated (i.e. driven well below their max parameters) [13]: we used two 0.35 Ω modules with I_max_ 9.2 A in a custom-made electrophysiology chamber holder, heat sinked with the aid of recirculating water (Fig. 6E).

**Fig. 4:**
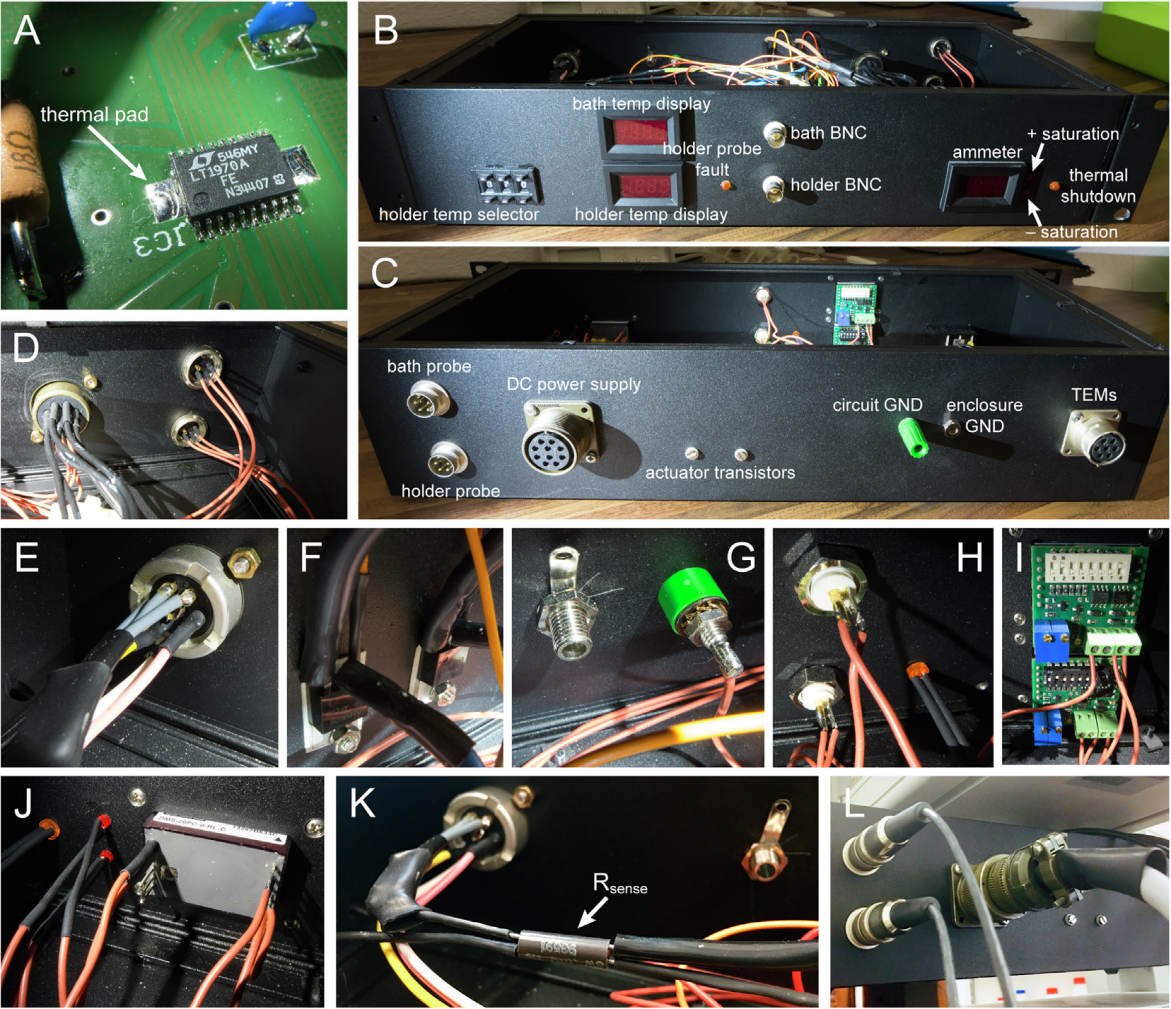
Backstage enclosure components and essential wiring diagram to the PCB. (A) Closeup on the power actuator chip, which requires soldering a bottom thermal pad. (B) Front plate. (C) Back plate. (D) DC power (left) and temperature probe receptacles (right). (E) TEM connector receptacle. (F) PNP and NPN power actuator transistors use the back plate as heat sink. (G) Enclosure (left) and circuit (right) grounding jacks. (H) Chamber holder and bath temperature output BNCs. (I) Chamber holder and bath temperature displays. (J) Ammeter. (K) Current sensing resistor. (L) Probe and power supply plugs connected to the backstage enclosure.

**Fig. 5:**
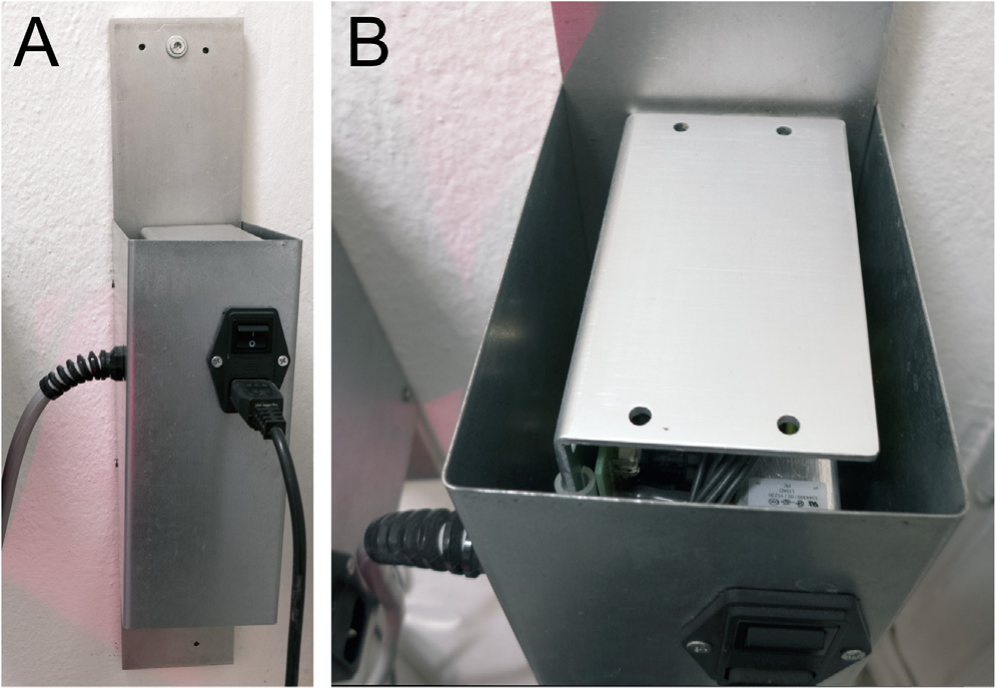
Possible solution for the remote power supply enclosure. (A) Front view: the aluminum plate attached to the wall acts as a heat sink; on the left is the DC cable going to the backstage enclosure, while on the front is the on/off switch with line filter and mains plug. (B) Top view.

**Fig. 6:**
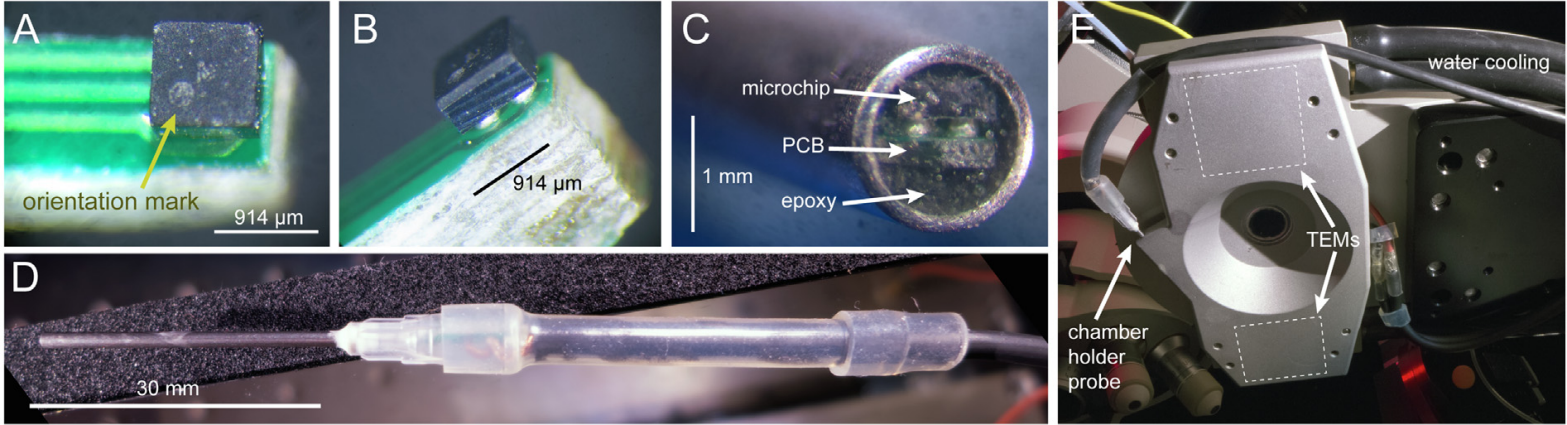
Temperature probe assembly. (A) The sensor microchip LMT70A (914x914x600 µm) must be soldered on the PCB in the correct orientation, indicated by the position of the package dot. (B) Two solder bumps can be seen below the package. (C) The microchip and PCB, once encased in transparent epoxy, should be visible at the tip of the needle. (D) The finished probe. (E) Custom made chamber holder for an upright microscope with two TEMs, water cooling and probe (seen from below).

Another important aspect is that of thermal expansion-dependent shifts in the preparation. In our experience one crucial factor in suppressing these movements is to avoid interposing any plastics between the TEMs and the tissue. One solution is to use a large diameter coverglass (e.g. 35 mm, #1.5) for the chamber bottom in direct contact with the holder aluminum. The holder should be designed so that the chamber bottom lies as near as possible to the plane of the TEMs in the *z*-axis. Furthermore, the chamber should sit in the geometric center of the TEMs in *x-y* (we used a pair of 30x30 mm TEMs; Fig. 6E). In our setup, for a 10 °C change in temperature we observe shifts in the preparation of < 1 µm in *z* and < 8 µm in *x-y*.

## 3 Design files

*modeling.cdf* — Public computable document format file with transfer functions, plots and graphs for the model described in 2.1, 2.4.1, 2.5. Modify parameters to predict your system’s response and to select an optimal $\tau_{c}$. Can be executed in Wolfram Mathematica® (fully functional 15-day free trial, download here) or Wolfram CDF Player (free, download here), both applications available for Windows, MacOS and Linux.

*backstage.sch*, *backstage.brd*, *probe.sch*, *probe.brd* — Eagle files of the schematics and boards of the backstage and temperature probe circuits. A free educational license of Eagle can be obtained at Autodesk if you wish to customize our design. The printed circuit boards (PCBs) can be purchased by uploading *backstage.brd* and *probe.brd* to any fabrication service (see *bill of materials.pdf*). These files can be also imported in the open source KiCad software starting from v.5.

backstage*.pdf, probe*.pdf —Views of the schematics and PCBs.

*backstage enclosure machining reference.dxf*, *backstage enclosure machining reference.dwg* — CAD files for drilling/milling hose and slots in the backstage aluminum enclosure.

*backstage enclosure machining reference.pdf* — Printable guide for manual drilling/milling holes and slots in the backstage aluminum enclosure.

*transfer function derivations.pdf* — derivations of the transfer functions of selected controller stages.

*wiring instructions.pdf* — list of the wiring connections between PCBs, power supply, connectors, cables.

List of design files

**Table:** 

Design file name	File type	Open source license	Location of the file
*modeling.cdf*	*Computable document format*	*CC BY 4.0*	*OSF repository*
*backstage.sch*	*Eagle schematic*	*CC BY 4.0*	*OSF repository*
*backstage.brd*	*Eagle board*	*CC BY 4.0*	*OSF repository*
*backstage schematic.pdf*	*pdf*	*CC BY 4.0*	*OSF repository*
*backstage pcb (transparent view, no GND plane).pdf*	*pdf*	*CC BY 4.0*	*OSF repository*
*backstage pcb (top view).pdf*	*pdf*	*CC BY 4.0*	*OSF repository*
*backstage pcb (bottom view).pdf*	*pdf*	*CC BY 4.0*	*OSF repository*
*probe.sch*	*Eagle schematic*	*CC BY 4.0*	*OSF repository*
*probe.brd*	*Eagle board*	*CC BY 4.0*	*OSF repository*
*probe schematic.pdf*	*pdf*	*CC BY 4.0*	*OSF repository*
*probe pcb (transparent view).pdf*	*pdf*	*CC BY 4.0*	*OSF repository*
*backstage enclosure machining reference.pdf*	*pdf*	*CC BY 4.0*	*OSF repository*
*backstage enclosure machining reference.dxf*	*dxf*	*CC BY 4.0*	*OSF repository*
*backstage enclosure machining reference.dwg*	*dwg*	*CC BY 4.0*	*OSF repository*
*bill of materials.pdf*	*pdf*	*CC BY 4.0*	*OSF repository*
*transfer function derivations.pdf*	*pdf*	*CC BY 4.0*	*OSF repository*
*wiring instructions.pdf*	*pdf*	*CC BY 4.0*	*OSF repository*

## 4 Bill of materials

Here we summarize the costs for each hardware element. Full details are provided in *bill of materials.pdf*.

This estimate excludes machine shop work (present in many academic institutions) and common lab tools: solder station, stereo microscope, heated stirrer. Significant cost savings can be obtained by choosing a cheaper backstage enclosure and in/out connectors. Further savings can be achieved by substituting the linear power supply with a high quality low noise switched supply.

Bill of materials summary

**Table:** 

Hardware element	Number of component types in bill of materials	Suggested component sellers	Approx. cost (excl. VAT)
*Backstage circuit*	*48*	*Mouser, Multi Circuit Boards*	*205 €*
*–characterizing components*	*for the actuator stage: Analog Devices LT1970A power Op Amp, ON Semi D44VH10G and D45VH10G bipolar transistors*		
*Backstage enclosure with temperature selector, meters, LEDs, transistors and connectors*	*18*	*Mouser, Digikey, Conrad, any machine shop*	*438 €*
*–characterizing components*	*for temperature selection: Bourns 3683S-1-103L three decades push button potentiometer*		
*Thermoelectric modules (TEM) with cable and connector*	*3*	*Mouser, Conrad*	*81 €*
*Linear power supply with cable and connectors*	*8*	*Mouser, Conrad, any machine shop*	*219 €*
*–characterizing components*	*International Power IHCC5-6/OVP linear supply*		
*Probe circuits*	*3*	*Mouser, Multi Circuit Boards*	*42 €*
*–characterizing components*	*Texas Instruments LMT70A sub-millimetric temperature sensor chip*		
*Probe enclosures with cables and connectors*	*4*	*Sigma-Merck, Conrad, Amazon*	*44 €*
***TOTAL***			***1029 €***

## 5 Build instructions

Refer to *bill of materials.pdf*, Fig. 1C and the PCB design files for the identity of each component.

### 5.1 Assembly of the backstage

*Step 1.* Solder the copper bottom plate of the power actuator chip (IC3 in *backstage pcb (transparent view, no GND plane).pdf*) to the heat sink pad on the PCB (Fig. 4A): cover the pad with no-clean flux paste, add a few mm of solder wire in small bits and put the chip on top (mind the package orientation dots); place the PCB on a lab stirrer and heat to ~ 200 °C; when the solder melts and spreads on the pad tap lightly on the chip while ensuring that all its pins are aligned with their respective pads; remove the PCB and let cool.
*Step 2.* Under a stereo microscope hand solder the pins of all surface mount chips using abundant no-clean flux (IC1-IC16; mind the package orientation dots): many instructional videos can be found on YouTube; clean flux with isopropanol; check that all pins make a contact to pads and not to each other.
*Step 3.* Solder all condensers and resistors to the PCB.
*Step 4.* Mount all components on the aluminum enclosure front/back plates (Fig. 1B and 4B,C; *backstage enclosure machining reference.pdf*, *wiring instructions.pdf*): holder temperature digital push-button selector (0.1 °C resolution) (4B), holder and bath temperature displays (4B,I), LEDs (4B,H,J), holder and bath temperature output BNCs (4B,H), ammeter (4B,J), temperature probe connector receptacles (4C,D right), DC power connector receptacle (4C,D left), power actuator transistors (4C,F), grounding jacks (4C,G), TEM connector receptacle (4C,E,K left), current sensing resistor R_sense_ (4 K right). Use wires of adequate cross section where necessary (Fig. 1B thick lines). Regarding the transistor pair, these form a classic push–pull power amplifier configuration together with the LT1970A (IC3) [8], [14] (Fig. 1B) and must be coupled to a heat sink: for simplicity and cooling efficiency we used the backstage chassis, which is made of aluminum (Fig. 4F). The desired temperature is coded as a linear signal, matched to the signal from the holder probe circuitry, and fed to the comparator (IC2) (Fig. 1B). This matching is obtained by simple scaling and shifting of the output of the temperature selector — a digital potentiometer that operates as a voltage divider — via precision voltage references (IC8, IC10) and amplifiers (IC9, IC11) (Fig. 1B). The signals from the two temperature probes are shifted, scaled and low pass filtered via a voltage reference (IC1) and amplifiers (IC4 or IC5). These are followed by unitary buffering stages (IC12 or IC15) that drive the two BNC outputs and a buffer (IC16) to interface with the comparator (Fig. 1B). A separate dual comparator (IC13) detects whether the signal from the holder probe is out of a realistic range (e.g. if is disconnected). In such a case it disables the power actuator chip (IC3) and signals the event by turning on a probe fault LED via a switch (IC7). The remaining components are described or referenced in other sections.

### 5.2 Assembly of the DC power supply

*Step 5.* Screw the linear power supply block to a heat sink; attach the line filter and the DC cable with its strain relief to a covering shield. Ensure good passive airflow (supply temperature should never exceed 50 °C) but prevent accidental access to internal components (Fig. 5).
*Step 6.* Connect the line filter and DC cable wires to the appropriate solder lugs on the power supply (*wiring instructions.pdf* and supply datasheet); attach the other end of the DC cable to the appropriate pins of the connector plug.

### 5.3 Assembly of the chamber holder and bath temperature probes

*Step 7.* Detach a probe PCB from the panelized board obtained from the factory, cover the 4 IC1 pads at its tip with a drop of no-clean flux, place a sensor microchip inside the drop and on the pads with the correct orientation (mind the orientation dot in *probe pcb (transparent view).pdf* and Fig. 6A); place the PCB tip on a lab stirrer and heat to ~ 200 °C while monitoring with a stereomicroscope; when the 4 solder bumps under the microchip melt it should spontaneously align with the pads (Fig. 6A,B); remove the PCB and let cool. Hand solder the miniature bypass capacitor C1 under a stereomicroscope, again with abundant flux.
*Step 8.* Use sandpaper to delicately shave off the bottom of the PCB until its entire shaft can be inserted in a 16G needle; leave the wider connector area at the back unsanded; solder connector pads X1-X3 to the probe cable wires (*wiring instructions.pdf*). Attach the other end of the cable (keep length below 3 m) to the appropriate pins of the connector plug.
*Step 9.* Test probe functionality by connecting it to the backstage enclosure and powering up the controller; the display should read an incorrect value that, however, changes with probe tip temperature.
*Step 10.* Draw silicone tubing over the PCB and cable; cut the needle so as match the PCB shaft length and sand the tip to a smooth surface; insert the PCB until the chip is just below the needle tip and embed in a small amount of transparent epoxy (Fig. 6C); after the resin sets slide the silicone tubing over the needle base (Fig. 6D).

If you need slightly a smaller probe tip you can design a probe PCB where one of the three traces runs on the bottom layer and have it fabricated at < 1 mm thickness; the narrow and thin profile should allow you to fit it in a 17G needle.

### 5.4 Two-point calibration of the temperature probe circuits

*Step 11.* On the back of the temperature displays set the DIP switches to: off,on,off,off,off,off,off,on (Fig. 4I); dip the entire length of a probe needle in a mixture of abundant crushed ice in deionized water (slush); on the back of the corresponding display adjust the left (offset) potentiometer until it reads “00.0”; now dip the probe tip in a stirred water bath set to 37.0 °C; adjust the right (span) potentiometer until the display reads “37.0”; check that the 0 °C calibration has not been affected (if necessary repeat this step). Note: check with a high accuracy thermometer with at least 0.1 °C resolution that both calibration solutions are at the correct temperatures.
*Step 12.* Repeat the calibration process for the second probe.

### 5.5 Calibration of the holder temperature selector circuit

Attach the two leads of a voltmeter to the JMP1 pad on the backstage PCB and any signal ground point.

*Step 13.* Set the temperature selector to “000”. Dip the holder probe in ice slush (the holder temp display should read “00.0”). Now turn the offset potentiometer R10 until the JMP1 voltage is zero (if the voltage swings are uncomfortably large or small, regulate these with potentiometer R17).
*Step 14.* Set the temperature selector to “370”. Dip the holder probe in a water bath at 37.0 °C (the holder temp display should read “37.0”). Now turn the gain potentiometer R9 until JMP1 voltage is zero. Check that the 0 °C calibration has not been affected by adjusting R9: it should not—if necessary repeat the calibration procedure a second time.

### 5.6 Assembly of the TEMs, chamber holder, cable and setting of the current limiter

*Step 15.* The specific assembly of the TEMs and chamber holder depend on the user configuration. Attach one end of the cable to the TEMs and the other to the connector plug (*wiring instructions.pdf*).
*Step 16.* On the backstage PCB bypass the controller stages $C_{2}\left(s\right),C_{3}\left(s\right)$ by running a wire between JMP1–JMP4; the system is now driven by the quasi-proportional controller $C_{1}\left(s\right)$ (Section 2.4.1): this is temporary to facilitate measuring parameters for the mathematical model.
*Step 17.* Thermally couple the holder probe to the chamber holder; turn on the system and select a temperature distant from ambient; one of the two saturation LEDs (Fig. 4B) should turn on: if not, increase the gain $K_{c}$ with potentiometer R17 and repeat; monitor the drive current on the ammeter and ensure that it falls within the limits specified in Section 2.7 by turning R22: this last step is necessary to ensure that the output current never exceeds the power supply specifications.

### 5.7 Estimate of model parameters $\tau_{s},t_{0},\tau_{d}$ (Eq. (3))

*Step 18.* Calibrate your A/D interface to convert the BNCs output to temperature (see Section 6) and monitor the chamber holder temperature; raise it by a few degrees from ambient, manually disconnect the TEM cable plug and record its passive decay; fit a single exponential and extract $\tau_{s}$.
*Step 19.* Also monitor the holder temperature selector circuit by hooking the central pin of a BNC cable to TP2 on the backstage PCB; connect the TEM cable and bring the holder temperature to your preferred working range (e.g. 37.0 °C); command a 0.5 °C step increase and record both signals; repeat after varying the controller gain with R17 until observing moderate ringing (Fig. 2A); measure the static error (the actual increase will be slightly<0.5 °C) and use it to estimate the open loop gain $K_{c}K$ (Section 2.4.1). Open *modeling.cdf* and replace your estimates of $\tau_{s}$ and $K_{c}K$ for 198 in *h[s_]* and 58 in *r1[s_]*; update the *r1* plot and compare it to your recorded response; iteratively adjust $t_{0}$ and $\tau_{d}$ in *d[s_]* starting from our default values 0.4 s and 5.5 s, until you match the actual responses (Fig. 2A) (it may be necessary to slightly adjust $K_{c}K$).

### 5.8 Estimate of model parameter $\tau_{c}$ (Eq. (5)) and completion of the controller

*Step 20.* In *modeling.cdf* iteratively update the root locus plot *p2* (Fig. 3C) while changing the value of $\tau_{c}$ in *c2[s_]* (our default is 3.167); select a compromise whereby the gain is as high as possible but all three roots are relatively close to the real axis and far to the left of the imaginary axis (Fig. 3A).
*Step 21.* Complete the $C_{2}\left(s\right)$ controller circuit by soldering identical resistors R14,R16 of value $\tau_{c}/3.4\mu \text{F}-412\Omega$ (Eq. (24) in *transfer function derivations.pdf*); remove the JMP1–JMP4 wire and connect JMP1–JMP2 and JMP3–JMP4.
*Step 22.* Bring the holder temperature to your preferred working range; command a 0.5 °C step increase while monitoring holder BNC output; increase the gain with R17 until you observe acceptable ringing (Fig. 2B). With a gain $K_{c}K$ of a few hundreds you will achieve a small static error.

## 6 Operation instructions

The apparatus is operational as soon as the power supply is switched on. The direction of current flow to the TEMs depends on whether the desired temperature, selected with a push-button digital potentiometer (0.1 °C resolution), is above or below that of the chamber holder. Given the high gain $K_{c}K$, as long as the two temperatures are significantly different the actuator stage will be in saturation, signaled by two LEDs (Fig. 4B). This is perfectly normal and simply informs the user that the max deliverable current is driving the TEMs, either in the heating (upper LED, + max current) or cooling direction (lower LED, – max current). This current is displayed by the ammeter. Since the feedback loop depends on the signal from chamber holder probe, the latter must be in thermal contact with the holder at all times or temperature runaway will occur. A holder probe fault LED warns when the probe is disconnected from the backstage enclosure or malfunctioning, or in any case when the measured temperature is outside of the range 0–46 °C. Two BNCs provide linearly scaled versions of holder and bath temperatures with an intercept of ~ 728 mV @ 0 °C and a slope of ~ 72.4 mV/ °C between 0 °C and 37 °C (for maximum accuracy the user should measure the BNC outputs while clamping the probes’ temperatures at each of the two calibration points). A thermal shutdown LED signals if the power actuator chip IC3 enters protection mode due to overheating: this will never happen when properly soldered (Fig. 4A).

### 6.1 Role of the bath probe

**Fig. 7:**
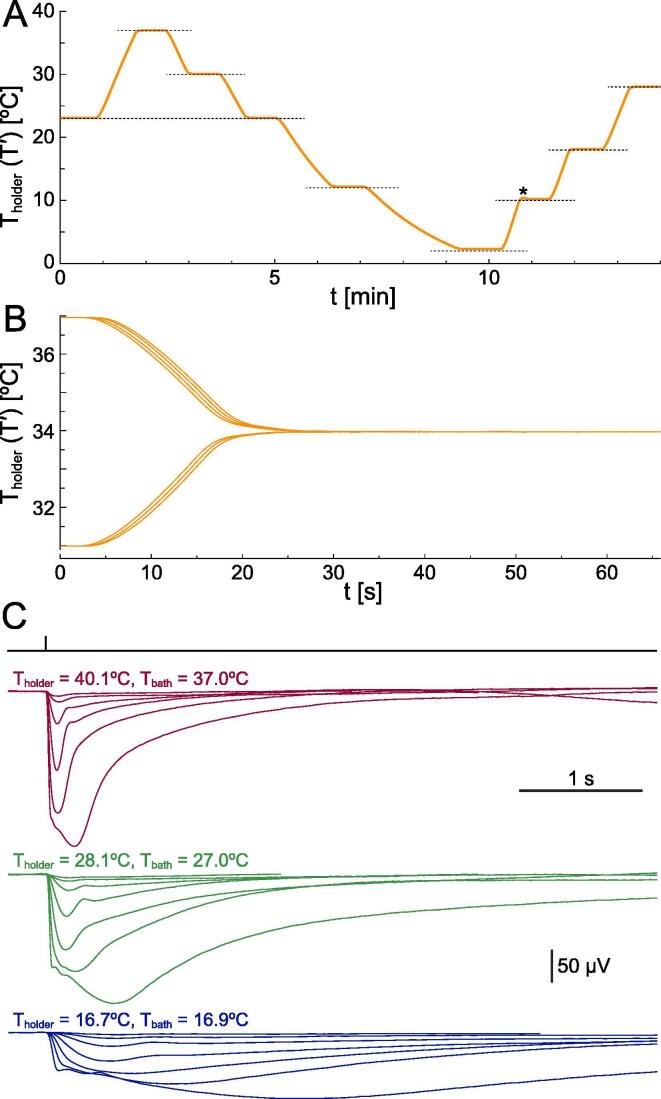
The temperature controller in actual use with our custom made chamber holder. (A) Response of the tissue chamber holder to large step changes in desired temperature: 23 → 37 → 30 → 23 → 12 → 2 → 10 → 18 → 28 °C (dashed lines). A slight overshoot is present only when increasing from very low levels (asterisk). The small amount of static error at low temperatures is due to the lower open loop gain when TEMs operate in cooling mode. If you only work in cooling mode you can optimize parameters accordingly. The trace was obtained via the holder probe BNC output. (B) Repeated changes from 37 °C or 31 °C to a desired temperature of 34 °C. Individual records are intentionally staggered in time to show the high reproducibility of trajectories. (C) *In vitro* electroretinogram recordings (DC–100 Hz) from the same dark adapted mouse retina showing the effect of temperature on flash response kinetics and amplitude. Tissue was kept in a custom 35 mm chamber during continuous superfusion of Ames’ medium (synaptic transmission to on-bipolars blocked with 40 µM DL-AP4). Full field light stimuli were delivered with a green LED. Dissection procedures and light stimulation details in [16]. Ambient temperature in all experiments was 23 °C. Note how the holder temperature was adjusted to obtain the final desired temperature at the preparation. (For interpretation of the references to colour in this figure legend, the reader is referred to the web version of this article.)

The bath probe is outside the feedback loop for reasons given in Section 2.7. Please note that nothing prevents the user from switching probes and attempting to clamp bath temperature, but we anticipate that stability will be hard to achieve. In our view the controller’s role is to fix the temperature around the tissue chamber. A temperature mismatch will exist between the holder and the bath (and often even within the bath), which will vary dramatically with holder and chamber design, superfusion flow rate, whether the incoming liquid is pre-conditioned, if objectives are being immersed or removed, etc. The bath probe is used for monitoring the preparation: it’s the experimenter’s responsibility to ensure that all these variables are kept constant so that a stable mismatch is maintained and the holder temperature set accordingly (Fig. 7C).

## 7 Validation and characterization

Much data on the performance of this system was already given in the previous sections. When coupled to a well designed tissue chamber holder (Fig. 6E) our controller can rapidly enact changes in temperature over a broad physiological range (Fig. 7A) — all without parameter adjustments other than setting the desired temperature. Temperature trajectories are highly reproducible and settle rapidly (Fig. 7B). Low noise electrophysiological recordings, both intracellular (e.g. patch clamp [15]) and extracellular (e.g. *ex vivo* electroretinography, Fig. 7C) can be made at single or multiple temperatures.

We examined the long term accuracy of the signal path from the probes to the displays, by dipping the chamber and bath probes next to each other in a vigorously stirred water bath. This was done three years after their most recent calibration had been performed (Section 5.4). In crushed ice and deionized water both displays read “00.0”, exactly as originally calibrated. In warm water at 37.0 °C (set with the same precision Hg thermometer originally used for calibration) the displays showed values within “00.1” of “37.0” and of each other. We conclude that long term accuracy is equal to or better than 0.1 °C. As discussed in Section 2.6, probes based on the LMT70A sensor and calibrated at 0 °C and 37 °C can be assumed to have linear output with a max inaccuracy of −0.04 °C at 18.5 °C. Linearity throughout the design range was confirmed to within the 0.1 °C resolution of a precision Hg thermometer.

The response kinetics of the probes were tested by transferring them from room temperature to an ice/water mixture (18 mm of their tips immersed) and found to be well described by the sum of two time constants with unequal weights: 0.58 s (91%) and 2.1 s (9%). If an even faster response is desired one may want to consider using thermally conductive epoxy in step 10 (Section 5.1).

With regards to the static error of the feedback loop, the system is free of hysteresis as it does not depend upon the direction from which the desired temperature is reached (Fig. 7B).

## 8 Suggestions for customization

Here we give suggestions to those users that might want to expand the performance envelope of this controller.

### 8.1 Increase output power

When one commands a change in desired temperature, the controller enters a regime of saturation whereby the TEMs are driven with the max voltage and current deliverable by the controller (Section 2.7). In this phase the TEMs pump roughly constant heat and the holder relaxes exponentially to a far off temperature with time constant $\tau_{s}$ (Section 2.1). As soon as the desired temperature is reached the controller interrupts the relaxation (these trajectories can be seen in Fig. 7A). Given a specific holder, the only way to hasten its temperature changes is to increase the controller power output. A relatively simple solution to obtain a + 50% increase in power would be to move to a linear supply with triple output + 5 V/+15 V/−15 V rated 3 A. The actuator stage amplifier LT1970A (IC3) and IC6, IC9, IC16 can handle ± 15 V rails, while the + 5 V rail would supply the LMT70A sensors, IC1, IC7, IC8, IC10, IC11, IC14 and the digital panel meters. The remaining chips IC2, IC4, IC5, IC12, IC15 would need a −5 V negative rail provided by a voltage regulator such as the LM7905. Some condensers and the TEMs would need to be upgraded to higher voltage ratings. If even more power is required, consider using a switching power supply with the same triple output but greater current capability. In any case, modifications in the output capabilities of the controller require careful selection of the TEMs to maximize heat pumping capacity: these should be driven roughly up to half their max rated current [13].

### 8.2 Add an analog external command input option

To programmatically control the temperature from a D/A converter, an elegant solution would be to piggyback on the calibrated circuit of the push-button selector (Section 5.5). The analog signal should be injected (under control of a new toggle switch on the backstage enclosure) at the point labelled ‘ext. comm.’ in Fig. 1B. Zero  °C would be coded by 0 V and higher temperatures with a scaling factor of 40.96 mV/ °C.

### 8.3 Implement separate open loop gains for heating and cooling

As mentioned in Section 2.1, TEMs are less efficient in cooling mode. Thus, their contribution to the open loop gain of the system will be lower and static error will be larger. To optimize system behavior through the entire 0–37 °C range one could compensate this TEM property by using a higher $K_{c}$ value when the controller is in the cooling polarity.

### 8.4 Adapt for use with heating only elements

In applications where the heating element is a resistance, bipolar operation must be prevented or the feedback loop will spin out of control. This can be achieved by inserting a power diode with a low forward voltage (e.g. ST Microelectronics STPS10L25D) in line with such element. We have successfully used our system in this mode to drive a silicone heating pad.

## 9 Animal rights

*In vitro* recordings were performed under approval by the ethical committee of the University of Pisa (prot. n. 2891/12) and conducted in accordance with Italian legislation (D.lgs.vo 116/92) and EU Directive 2010/63/EU for animal experiments. Retinas from adult wild type C57Bl/6J mice were isolated as previously described [16].

## Declaration of Competing Interest

The authors declare that they have no known competing financial interests or personal relationships that could have appeared to influence the work reported in this paper.
