# Supplementary material for: Versatile bipolar temperature controller for custom in vitro applications
Source: HardwareX. 2020 Nov 3;8:e00155. doi: 10.1016/j.ohx.2020.e00155 (PMC7874220; doi:10.1016/j.ohx.2020.e00155)
Supplement: Supplementary data 1 [file mmc1.pdf]

All design files have been uploaded to the OSF repository and can be found at this link: Open Science Framework (OSF)
